# Impaired circumferential and longitudinal myocardial deformation in early stage chronic kidney disease: the earliest features of uremic cardiomyopathy

**DOI:** 10.1186/1532-429X-15-S1-P153

**Published:** 2013-01-30

**Authors:** Nicola C Edwards, Abdullah Noori, Colin D Chue, William E Moody, Charles J Ferro, Jonathan N Townend, Richard Steeds

**Affiliations:** 1 Cardiovascular Medicine, University of Birmingham & Queen Elizabeth Hospital Birmingham, Birmingham, UK; 2 Cardiology, Queen Elizabeth Hospital Birmingham, Birmingham, UK; 3 Cardiovascular Medicine, University of Birmingham & Queen Elizabeth Hospital Birmingham, Birmingham, UK; 4 Cardiovascular Medicine, University of Birmingham & Queen Elizabeth Hospital Birmingham, Birmingham, UK; 5 Nephrology, University of Birmingham & Queen Elizabeth Hospital Birmingham, Birmingham, UK; 6 Cardiology, University of Birmingham & Queen Elizabeth Hospital Birmingham, Birmingham, UK; 7 Cardiology, University of Birmingham & Queen Elizabeth Hospital Birmingham, Birmingham, UK

## Background

There is a graded inverse relationship from early stages of chronic kidney disease (CKD) (GFR<75ml/min/1.73m2) with increased cardiovascular risk that is driven by high rates of heart failure and sudden cardiac death. Abnormal left ventricular (LV) deformation is an independent predictor of poor prognosis in end-stage CKD and is thought to reflect the extensive interstitial fibrosis and myocyte disarray documented on LV biopsy in uremic cardiomyopathy (UC). There remains a paucity of data relating to the onset of UC in early stage CKD.

## Methods

Eighteen patients (mean age 51 +/- 15 years) with non-diabetic stage 3 CKD (mean eGFR 50 +/- 16 ml/min/1.73m2 MDRD) were compared with 18 healthy age and sex matched controls. Patients all had well controlled blood pressure (mean 24 hour BP 123 ± 14 / 73 ± 6 mmHg) on an average of 1.1 antihypertensive agents and had no history of cardiovascular disease. Cardiac MRI (1.5T Siemens Avanto) with (SPAMM) tagging was performed. Data was analysed using commercially available software (CIM-2DTag, University of Auckland) to assess global and transmural LV deformation at SAX basal, mid, apical and long-axis HLA levels.

## Results

Longitudinal and circumferential strain (S) increased in a graded fashion from epicardium (epi) to mid-wall (mw) to endocardium (endo) at basal, mid and apical ventricular levels in both CKD and controls. Global basal circumferential S (-13.3% +/- 2.3 vs. -15.9% + / - 2.9, p<0.05) and strain rate (SR) (0.7 +/- 0.4s-1 vs. -1.1 +/- 0.6s-1, p<0.05) were reduced in CKD. Basal circumferential S and SR at epi, mw and endo levels were reduced but there was no transmural difference at mid and apical ventricular levels (Table 1). Global longitudinal LV strain (-14.2% +/- 1.7 vs. -15.9% +/- 2.3, p<0.05) and strain rate (0.69 +/- 0.1s-1 vs. 0.84 +/- 0.1s-1, p<0.05) were reduced in CKD and at mw and endo levels compared to controls (Table 1). There was no difference in conventional measures of LV EF, LV mass or volumes (Table 1).

**Table 1:** Parameters of left ventricular systolic function

		CKD	Controls		CKD	Controls
SAX:Base	Epi S	-13.3 +/- 2.3†	-15.9 +/- 2.9	Epi SR	-1.1 +/- 0.4†	-1.4 +/- 0.5

	MW S	-13.0 +/- 2.3†	-15.6 +/- 2.9	MW SR	-0.7 +/- 0.4†	-1.4 v 0.5

	Endo S	-18.1 +/- 3.2†	-21.0 +/- 4.4	Endo SR	-0.5 +/- 0.2†	-1.1 +/- 0.2

SAX:Mid	Epi S	-13.3 +/- 2.0	-13.1 +/- 2.0	Epi SR	-1.6 +/- 0.5	1.4 +/- 0.6

	MW S	-19.0 +/- 2.1	-18.0 +/- 2.1	MW SR	-1.1 +/- 0.4	-1.2 +/- 0.4

	MW S	-25.2 +/- 2.5	-24.0 +/- 2.1	Endo SR	-1.0 +/- 1.1	-1.2 +/- 0.2

SAX:Apex	Epi S	-13.8 +/- 1.7	-14.4 +/- 2.3	Epi SR	-1.6 +/- 0.7	-1.1 +/- 0.7

	MW S	-19.9 +/- 1.9	-19.6 +/- 1.7	MW SR	-1.0 +/- 0.4	-1.0 +/- 0.4

	Endo S	-28.2 +/- 2.8	-27.7 +/- 2.9	Endo SR	-1.3 +/- 0.3	-1.4 +/- 0.5

LAX	Epi S	-12.7 +/- 1.7†	-14.7 +/- 2.9	Epi SR	0.7 +/- 0.1	-0.8 +/- 0.1

	MW S	-14.1 +/- 1.7†	-16.1 +/- 2.1	MW SR	-0.8 +/- 0.2	-0.97 +/- 0.1†

	Endo S	-15.4 +/- 2.8†	-17.8 +/- 2.0	Endo SR	-0.9 +/- 0.1	-1.09 +/- 0.1†

LV Mass (g/m2)		57 +/- 22	59 +/- 11			

LVEF (%)		74 +/- 7	75 +/- 5			

† p < 0.05 Epi; epicardium, MW; mid-wall, Endo; endocardium, EF; ejection fraction

## Conclusions

The earliest systolic abnormalities of UC are altered circumferential basal S and SR and reduced longitudinal function. These parameters might serve as early imaging biomarkers that could be used to activate pharmacological intervention. The circumferential abnormalities differ to those observed with aging, hypertension and HFPEF, suggesting a different pathophysiology and necessitate future studies to address whether focal changes in myocardial interstitial fibrosis are responsible for these changes in deformation.

## Funding

British Heart Foundation FS/11/17/28700, PG /04/109

